# Tobacco Susceptibility Explains Diminished Returns of Family Income on Black Adolescents’ Tobacco Initiation

**DOI:** 10.31586/ojp.2024.1037

**Published:** 2024-08-27

**Authors:** Shervin Assari, Payam Sheikhattari

**Affiliations:** 1 Department of Internal Medicine, Charles R. Drew University of Medicine and Science, Los Angeles, CA, United States; 2 Department of Family Medicine, Charles R. Drew University of Medicine and Science, Los Angeles, CA, United States; 3 Department of Urban Public Health, Charles R. Drew University of Medicine and Science, Los Angeles, CA, United States; 4 Marginalization-Related Diminished Returns (MDRs) Center, Los Angeles, CA, United States; 5 The Prevention Sciences Research Center, School of Community Health and Policy, Morgan State University, Baltimore, MD, USA; 6 Department of Behavioral Health Science, School of Community Health and Policy, Morgan State University, Baltimore, MD, USA

**Keywords:** Family Income, Tobacco Susceptibility, Black Adolescents, Tobacco Initiation, Health Disparities, ABCD Study

## Abstract

**Background:**

Minorities’ Diminished Returns (MDRs) theory posits that socioeconomic resources have weaker protective effects on health and behavior for racial and ethnic minorities compared to Whites. This study examines whether tobacco susceptibility, defined as curiosity, intention, and openness to future tobacco use, mediates the diminished returns of family income on tobacco initiation among Black adolescents.

**Methods:**

Data from the Adolescent Brain Cognitive Development (ABCD) Study were analyzed. Participants were followed from age 9 to 16. All participants were tobacco naive at baseline. Tobacco susceptibility was assessed through self-reported measures of curiosity, intention, and openness to future tobacco use. Structural equation modeling (SEM) was used to examine the relationship between family income, tobacco susceptibility, and tobacco initiation.

**Results:**

Overall, 10,653 Black or White youth entered our analysis. The analysis revealed that higher family income was less effective in preventing tobacco initiation among Black adolescents. Tobacco susceptibility significantly predicted tobacco initiation and partially mediated the relationship between family income and tobacco initiation.

**Conclusions:**

Tobacco susceptibility explains some of the diminished returns of family income on tobacco initiation among Black adolescents. Interventions aimed at reducing tobacco susceptibility may enhance the protective effects of family income and help mitigate health disparities.

## 1. Introduction

Minorities’ Diminished Returns (MDRs) [1, 2] is a theoretical framework that explains why socioeconomic resources, such as income and education, yield fewer health and behavioral benefits for racial and ethnic minorities compared to Whites. These diminished returns are primarily driven by systemic and structural racism [3–6]. For instance, segregation in housing and education limits access to quality resources and safe environments, reducing the benefits typically associated with higher income [7–9]. Similarly, labor market discrimination restricts opportunities for economic mobility, further weakening the protective effects of income for minority families [10, 11]. These disparities are deeply embedded in societal structures, perpetuating inequities across generations.

Previous research has demonstrated that higher family income is less effective in preventing tobacco use initiation among Black adolescents compared to their White counterparts [12]. This diminished protective effect persists despite similar levels of socioeconomic resources among Black families [5, 13–17]. Environmental stressors [18], neighborhood conditions [19], and differential exposure to advertising and peer influences [20] contribute to this phenomenon, reflecting broader societal inequities.

Tobacco susceptibility, defined as curiosity, intention, and openness to future tobacco use, is a critical predictor of tobacco initiation among adolescents [21, 22]. The literature consistently shows that adolescents who exhibit higher levels of curiosity, intention, and openness to future tobacco use are more likely to initiate use in the future [23, 24]. This susceptibility is influenced by various factors, including peer influence, media exposure, and perceived social norms around tobacco use [25]. Despite the established link between tobacco susceptibility and initiation, it remains unclear whether tobacco susceptibility explains the diminished returns of family income on future tobacco use among Black adolescents [26–29]. Understanding this relationship is crucial, as it may highlight specific intervention points to reduce tobacco initiation in this vulnerable population.

## 2. Aims

Built on the Minorities’ Diminished Returns (MDRs) theory [5, 30], this study aims to investigate whether tobacco susceptibility mediates the weaker protective effects of family income on tobacco initiation among Black adolescents. We hypothesize that higher tobacco susceptibility will explain, at least in part, the diminished returns of family income on preventing tobacco use in this group.

## 3. Methods

### 3.1. Design and Setting

This study conducted a secondary analysis of the Adolescent Brain Cognitive Development (ABCD) study [31], a comprehensive and advanced investigation into the neurocognitive mechanisms associated with the onset of substance use among children transitioning to emerging adulthood. The ABCD study is longitudinal, collecting data on substance use every six months, with major data collection waves occurring every two years. Detailed descriptions of the ABCD study’s methods are available in other publications [31].

### 3.2. Sample and Sampling

The ABCD study initially recruited over 11,000 children aged 9 to 10 years from 21 sites in 19 cities across 15 states in the United States. Although the sample was not randomly selected, it closely matches the demographic characteristics of U.S. children in this age group during 2016-2018. The primary sampling frame for the ABCD study was U.S. schools.

### 3.3. Analytical Sample

For this analysis, the sample included 10,653 children who had follow-up data over six years regarding their tobacco use as they transitioned to early and middle adolescence. Inclusion criteria for this study were being between 9 and 10 years old at baseline, being tobacco naïve at baseline, having some follow-up data on tobacco use, and being either Black or White.

### 3.4. Variables

#### 3.4.1. Moderator

##### Race:

Self-identified race was categorized as White (0) or Black (1). Participants identifying as Asian, mixed, or other races were excluded.

#### 3.4.2. Mediator

##### Tobacco Susceptibility:

Assessed using a three-item measure evaluating future use intention, openness to use, and curiosity. The measure had a baseline Cronbach’s alpha slightly below 0.5, which improved in subsequent waves. It was treated as a continuous variable, with higher scores indicating greater susceptibility to tobacco use [31].

#### 3.4.3. Predictor

##### Family Income:

Family income was measured on a 1-10 scale, reflecting total combined family income over the past 12 months, with categories ranging from less than $5,000 to $200,000+.

#### 3.4.4. Outcome

##### Tobacco Use:

Tobacco use was measured using the iSay Sipping Inventory and a web-based Timeline Follow-Back (TLFB) method. Baseline assessments captured lifetime substance use, and follow-up assessments at six-month intervals captured substance use since the last study session. For this analysis, substance use was defined as experimentation with tobacco (puffing) and initiation of tobacco use (more than one puff) [31].

#### 3.4.5. Covariates

##### Age:

Reported in months, calculated from birth to study enrollment.

##### Parental Education:

Reported by parents as years of schooling, operationalized as a continuous variable based on the Jaeger scale.

##### Family Structure:

Categorized based on the number of parents in the household and their marital status, coded as 0 for non-married households and 1 for married households.

##### Sex:

Dichotomous variable coded as males (1) and females (0).

### 3.5. Statistical Analysis

Data analysis was performed using SPSS. Descriptive statistics included means and standard deviations for continuous variables and frequencies for categorical variables. Pearson correlations were calculated for bivariate associations among study variables. Multivariable modeling involved four Structural Equation Models (SEMs) with subsequent tobacco use as the outcome, household income as the predictor, race as the moderator, and sex, age, family structure, and parental education as covariates. Tobacco susceptibility was the mediator. Model significance was set at the 0.001 level, and results were reported with unstandardized coefficients (b), standard errors (SE), 95% confidence intervals (CI), and p-values, with p ≤ 0.05 considered significant.

### 3.6. Ethics

The ABCD study received Institutional Review Board (IRB) approval from multiple institutions, including the University of California, San Diego (UCSD). All participants provided assent, and their parents gave informed consent. This secondary analysis utilized fully de-identified data, classifying it as non-human subject research, and was therefore exempt from a full IRB review.

## 4. Results

Overall, 10,653 Black or White youth entered our analysis. Table 1 shows the description of study variables. Most families were married, and almost half of the sample was male. Slightly more than 5% showed subsequent tobacco use.

Table 2 shows correlations between study variables. While marital status, parental education, and household income were correlated with each other, these variables showed weak or no correlation with subsequent tobacco use. Tobacco susceptibility was positively correlated with subsequent tobacco use.

**Figure 1.**
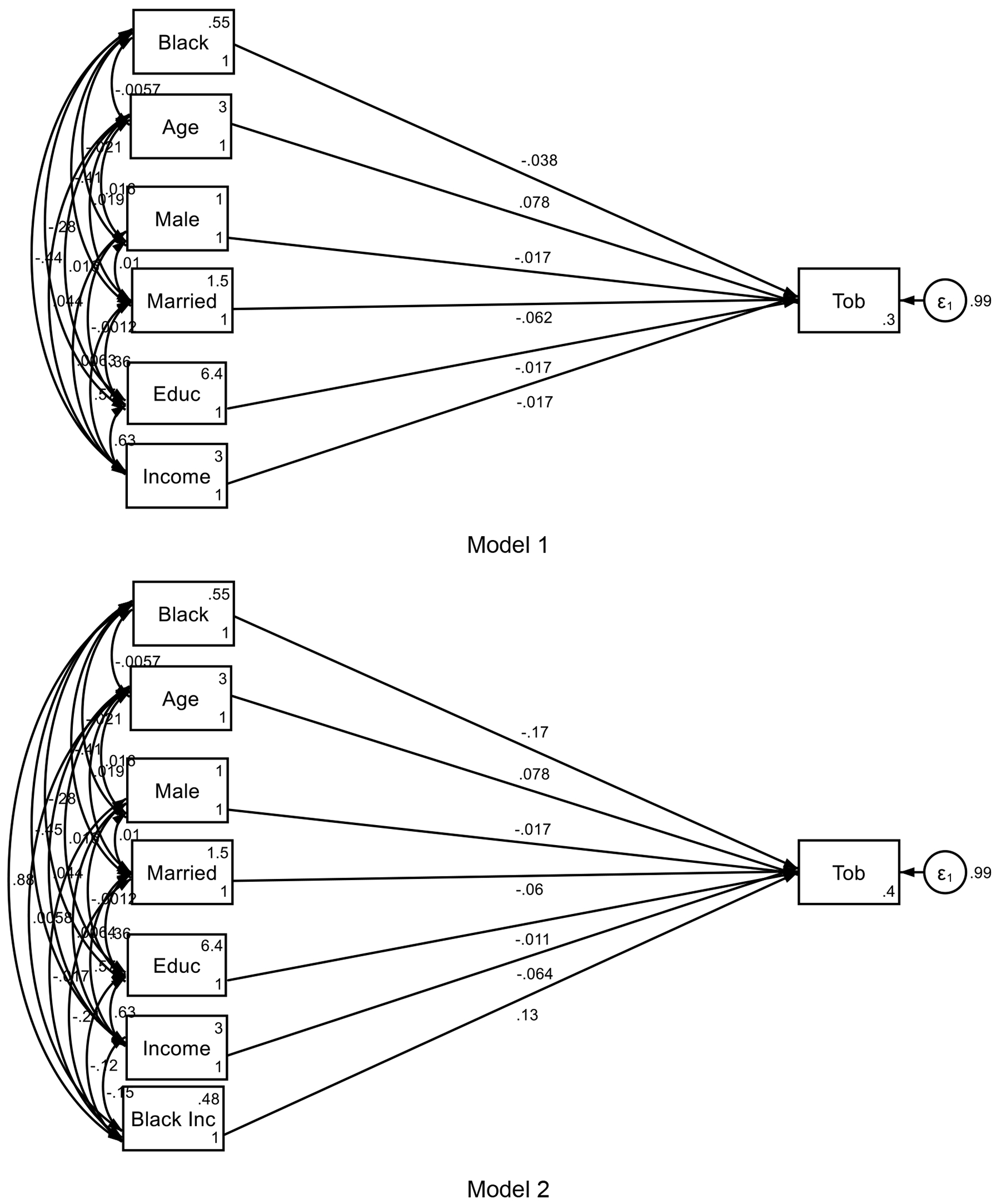

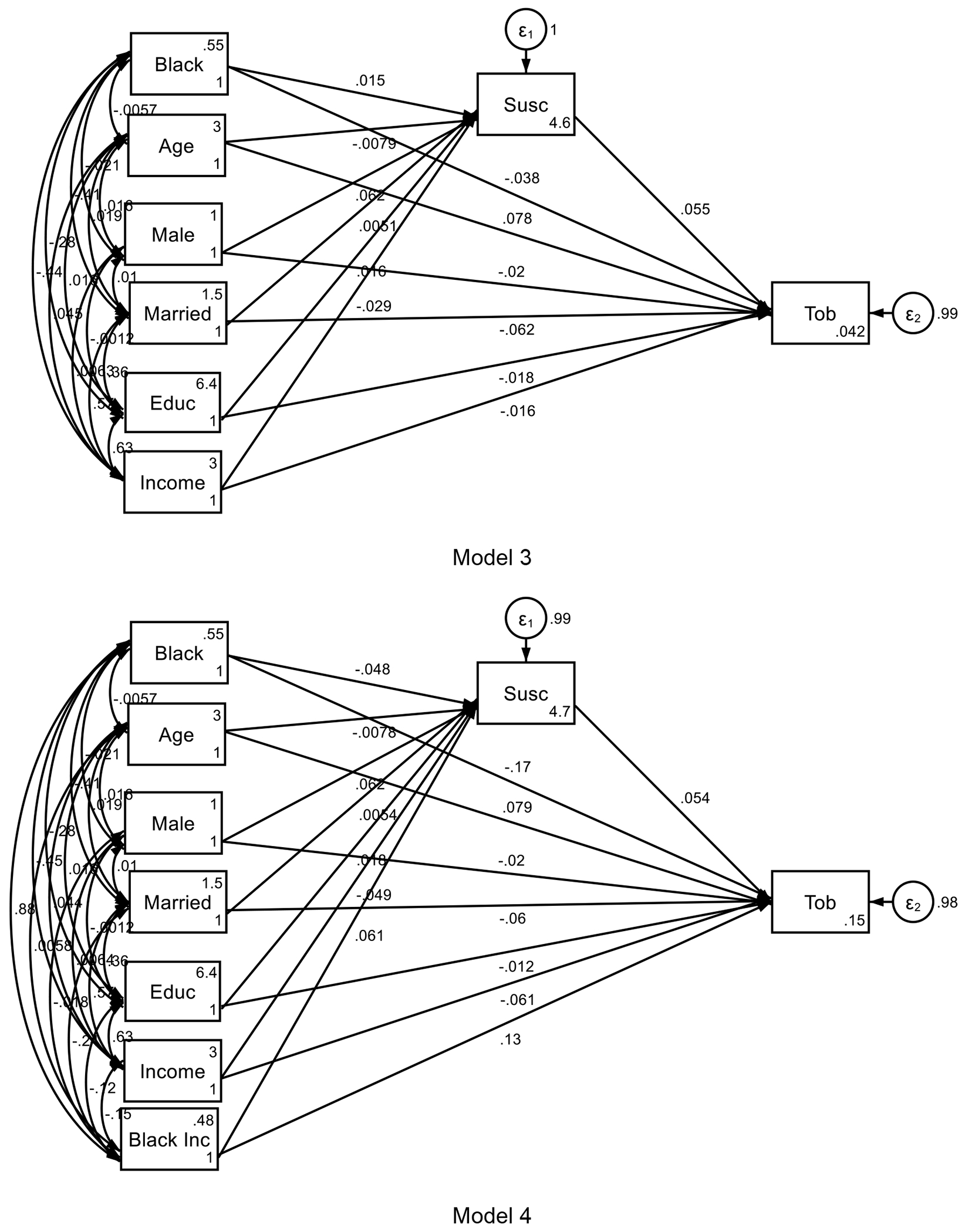
Summary of Structural equation models (SEMs)

Table 3 and Figure 1 shows the summary of our Model 1 to Model 4. The analysis revealed that higher family income was less effective in preventing tobacco initiation among Black adolescents. Tobacco susceptibility significantly predicted tobacco initiation and partially mediated the relationship between family income and tobacco initiation.

## 5. Discussion

This longitudinal study investigated the relationship between family income, tobacco susceptibility, and tobacco initiation among Black and White adolescents using data from the Adolescent Brain Cognitive Development (ABCD) Study. Our findings indicate that tobacco susceptibility mediates the differential relationship between family income and youth tobacco use in Black and White populations. In other words, higher tobacco susceptibility explains why Black youth from higher-income families remain at risk of tobacco use, while high-income White youth are more protected against tobacco initiation.

The Minorities’ Diminished Returns (MDRs) theory [19, 32–34] posits that the benefits of socioeconomic resources, such as income, tend to be attenuated for racial and ethnic minorities due to systemic discrimination, structural racism, social stratification, and segregation. Previous research has shown that factors like residential segregation [19], school inequalities [20], and labor market discrimination [35] contribute to the diminished returns of SES indicators for Black families by limiting access to quality resources and opportunities. These structural barriers perpetuate health and behavioral disparities, reinforcing the cycle of disadvantage for minority populations [36–39].

Our study adds to the understanding of the mechanisms underlying MDRs in the context of tobacco use [40]. We identified disproportionately high levels of tobacco susceptibility as a psychological mechanism explaining the weaker protective effects of family income on tobacco initiation among Black adolescents. Higher levels of curiosity, intention, and openness to future tobacco use among Black adolescents may stem from differential exposure to pro-tobacco influences that are not mitigated by higher family income [29]. Black communities are often targeted by predatory marketing from the tobacco industry, and retail density of tobacco products is higher in predominantly Black areas, which is a predictor of tobacco use [41–49].

This finding underscores the importance of addressing individual-level psychological factors in conjunction with broader structural determinants to effectively reduce health disparities [50, 51]. Educational programs targeting Black and low SES youth could be an effective way to prevent tobacco use in these communities [52]. Structural racism and social stratification play pivotal roles in reducing the returns of income for Black families [5, 53–56]. Across levels of family income, Black adolescents face significant barriers, including exposure to tobacco marketing, limited access to preventive health services, and higher levels of community violence and stress. These factors can diminish the protective effects of socioeconomic resources, making it more challenging for Black families to achieve the same health and behavioral outcomes as their White counterparts.

Regardless of family income, Black adolescents often reside in environments with higher levels of risk factors, including peer influences that promote tobacco use [57]. Family risk of tobacco use is also higher in high SES Black families [58], who may also be exposed to secondhand tobacco smoke [59, 60]. These high-risk environments can negate the benefits of higher family income by exposing Black adolescents to more significant social pressures and opportunities to engage in tobacco use. This highlights the need for community-level interventions that target environmental and social determinants of health [61–64].

### 5.1. Policy Implications

The findings of this study have critical implications for public health policy and prevention efforts. Interventions aimed at reducing tobacco susceptibility among Black adolescents should be prioritized, including programs that address curiosity, intention, and openness to future tobacco use. Additionally, policies that address structural determinants of health, such as reducing segregation and improving access to quality education and healthcare, are essential to enhance the protective effects of family income. If tobacco susceptibility is found to mediate the diminished returns of family income, targeted interventions addressing curiosity, intention, and openness to future tobacco use may be necessary to enhance the protective effects of socioeconomic resources. Addressing broader structural issues such as segregation and discrimination remains essential to reduce the overall burden of tobacco use and its related health disparities. This study contributes to the understanding of Minorities’ Diminished Returns (MDRs) by highlighting a potential psychological mechanism through which these diminished returns operate. Future research should continue to explore other mediating factors and develop comprehensive strategies that address both individual and structural determinants of health. Comprehensive strategies that combine individual and structural approaches are necessary to reduce tobacco use and health disparities among Black adolescents.

### 5.2. Future Research

Future research should continue to explore the mechanisms underlying MDRs that increase tobacco use among high SES Black youth, particularly focusing on psychological and environmental factors that may mediate the relationship between socioeconomic resources and health outcomes. Longitudinal studies that follow adolescents into adulthood can provide valuable insights into the long-term effects of these mechanisms. Additionally, research should investigate the effectiveness of targeted interventions and policies in reducing tobacco susceptibility and improving health outcomes for minority populations. As this study only included Black and White populations, future research should also test similar patterns for other marginalized groups, such as Latino populations.

### 5.3. Limitations

This study has several limitations that should be acknowledged. First, the self-reported measures of tobacco susceptibility and use may be subject to reporting biases. Second, the study’s observational design with few control variables limits our ability to draw causal conclusions. The follow-up period was relatively short, and only a few youth initiated tobacco use during this time. Despite these limitations, the study provides valuable insights into the mechanisms underlying MDRs and highlights important areas for future research and intervention.

## 6. Conclusion

This study demonstrates that tobacco susceptibility partially mediates the diminished returns of family income on tobacco initiation among Black adolescents. These findings underscore the need for targeted interventions that address both individual-level psychological factors and broader structural determinants of health. By addressing these factors, policymakers and public health practitioners can develop more effective strategies to reduce tobacco use and health disparities among Black adolescents.

## Figures and Tables

**Table 1.** Descriptive Data (n=10,653)

	Mean	SD
HH Income	7.42	0.02
Parent Education	16.99	0.03
Tobacco Susceptibility	1.08	0.00
	N	%
Race (Black)		
White	8,171	76.70
Black	2,482	23.30
Household Marital Status		
Not Married	3,388	31.80
Married	7,265	68.20
Sex		
Female	5,101	47.88
Male	5,552	52.12
Age		
9 Yr	5,582	52.40
10 Yr	5,071	47.60
Subsequent Tobacco Use		
No	10,004	94.92
Yes	535	5.08

**Table 2.** Bivariate Correlations (n = 10,653)

	1	2	3	4	5	6	7	8
1 Race (Black)	1.000							
2 Married HH	−0.410*	1.000						
3 Sex (Male)	−0.023	0.014	1.000					
4 Age (10)	−0.005	0.019	0.016	1.000				
5 HH income	−0.437*	0.558*	0.006	0.042*	1.000			
6 Parent Education	−0.285*	0.357*	0.000	0.018	0.617*	1		
7 Tobacco Susceptibility	0.019	−0.011	0.061*	−0.008	−0.023	−0.0049	1	
8 Subsequent Tobacco Use	−0.003	−0.057	−0.020	0.076*	−0.041*	−0.0382	0.053*	1.000

* p < 0.05

**Table 3.** Summary of Structural equation modeling (SEMs) (n = 10,653)

	Beta	std. err.	[95% conf. interval]		P>z
**Model 1**					
Subsequent Tobacco Use					
Parental Education	−0.017	0.013	−0.042	0.008	0.179
HH Income	−0.017	0.015	−0.047	0.012	0.254
Black	−0.038	0.011	−0.059	−0.016	0.001
Age (10)	0.078	0.010	0.059	0.097	< 0.001
Male	−0.017	0.010	−0.036	0.002	0.084
Married HH	−0.062	0.012	−0.085	−0.038	< 0.001
Intercept	0.298	0.074	0.153	0.443	< 0.001
					
**Model 2**					
Subsequent Tobacco Use					
Parental Education	−0.011	0.013	−0.035	0.014	0.399
HH Income	−0.064	0.017	−0.098	−0.030	< 0.001
Black	−0.171	0.028	−0.226	−0.116	< 0.001
Age (10)	0.078	0.010	0.059	0.097	< 0.001
Male	−0.017	0.010	−0.036	0.002	0.078
Married HH	−0.060	0.012	−0.083	−0.036	< 0.001
Black x HH Income	0.130	0.025	0.081	0.179	< 0.001
Intercept	0.405	0.077	0.255	0.555	< 0.001
					
**Model 3**					
Tobacco Susceptibility					
Parental Education	0.016	0.013	−0.010	0.042	0.239
HH Income	−0.029	0.016	−0.061	0.002	0.071
Black	0.015	0.012	−0.008	0.038	0.204
Age (10)	−0.008	0.010	−0.027	0.012	0.431
Male	0.062	0.010	0.043	0.082	< 0.001
Married HH	0.005	0.013	−0.020	0.030	0.687
Intercept	4.605	0.085	4.438	4.773	< 0.001
					
Subsequent Tobacco Use					
Tobacco Susceptibility	0.055	0.010	0.036	0.075	< 0.001
Parental Education	−0.018	0.013	−0.043	0.007	0.156
HH Income	−0.016	0.015	−0.045	0.014	0.308
Black	−0.038	0.011	−0.060	−0.017	< 0.001
Age (10)	0.078	0.010	0.059	0.097	< 0.001
Male	−0.020	0.010	−0.039	−0.001	0.037
Married HH	−0.062	0.012	−0.086	−0.038	< 0.001
Intercept	0.042	0.087	−0.129	0.214	0.627
					
**Model 4**					
Tobacco Susceptibility					
Parental Education	0.018	0.013	−0.008	0.044	0.182
HH Income	−0.049	0.018	−0.085	−0.013	0.008
Black	−0.048	0.030	−0.108	0.011	0.109
Age (10)	−0.008	0.010	−0.027	0.012	0.438
Male	0.062	0.010	0.043	0.082	< 0.001
Married HH	0.005	0.013	−0.019	0.030	0.670
Black x HH Income	0.061	0.027	0.008	0.115	0.023
Intercept	4.658	0.088	4.486	4.831	< 0.001
					
Subsequent Tobacco Use					
Tobacco Susceptibility	0.054	0.010	0.034	0.074	< 0.001
Parental Education	−0.012	0.013	−0.036	0.013	0.355
HH Income	−0.061	0.017	−0.095	−0.027	< 0.001
Black	−0.168	0.028	−0.223	−0.113	< 0.001
Age (10)	0.079	0.010	0.060	0.097	< 0.001
Male	−0.020	0.010	−0.039	−0.001	0.035
Married HH	−0.060	0.012	−0.084	−0.037	< 0.001
Black x HH Income	0.127	0.025	0.077	0.176	< 0.001
Intercept	0.152	0.090	−0.024	0.328	0.091
